# HIV and sexually transmitted infections: reconciling estranged bedfellows in the U = U and PrEP era

**DOI:** 10.1002/jia2.25357

**Published:** 2019-08-30

**Authors:** Kenneth H Mayer, Henry JC de Vries

**Affiliations:** ^1^ The Fenway Institute Fenway Health Department of Medicine Beth Israel Deaconess Medical Center MA USA; ^2^ Department of Medicine Harvard Medical School Boston MA USA; ^3^ Amsterdam UMC Department of Dermatology Location Academic Medical Center Amsterdam Institute for Infection and Immunology (AI&II) University of Amsterdam Amsterdam Netherlands; ^4^ Department of Infectious Diseases Public Health Service Amsterdam Amsterdam The Netherlands

**Keywords:** HIV, STI, PrEP, primary prevention, secondary prevention, sexual behaviour

Since the earliest days of the AIDS epidemic, it was clear that HIV and other sexually transmitted infections (STI) had many features in common 1. Their spread involved the same behaviours, and often affected the same socially marginalized people, including men who have sex with men (MSM), sex workers, substance users and migrants. As the aetiologic agent of AIDS, HIV, was elucidated, it became clearer that there were biological interactions between HIV and STI. Inflammatory and ulcerative STI facilitated HIV transmission and acquisition, and HIV infection led to increased infectiousness of several STI pathogens 2, 3. A more sophisticated understanding of their epidemiology also suggested that individuals who were engaging in behaviours that led to STI acquisition were more likely to be part of sexual networks where HIV transmission or acquisition were more likely. Prior to the advent of antiretrovirals for prevention, common strategies employed to decrease HIV/STI spread, mainly involved the promotion of “ABC”: Abstinence, or Behaviour change (decreasing the number of partners), and Condom use.

However, over the past decade, the evidence proving that people living with HIV who had undetectable plasma HIV RNA do not transmit HIV (U = U) 4, 5, and the demonstration that pre‐exposure prophylaxis (PrEP) using tenofovir‐based regimens protects individuals against the sexual acquisition of HIV 6, 7, 8 has altered the dynamics of HIV‐STI epidemiologic synergy. In the current era, individuals who are adherent to antiretroviral medication, whether for treatment or prevention, can expect to engage in condomless intercourse without either acquiring HIV or transmitting the virus to others, but are still at high risk for acquiring and transmitting STI. Moreover, during this same time period, STI increases have been occurring globally, particularly in key populations.

Historically, HIV researchers, STI specialists and frontline clinicians have had differing perspectives about the relationships between HIV and STI. In the earliest days of the epidemic, HIV research was focused on trying to identify an unknown, highly lethal pathogen, whereas most STI pathogens were well known and well described. HIV clinical care involved treating individuals who were at risk for recurrent opportunistic infections and neoplasms, whereas STI management was able to focus on the development of systems to diagnose and treat infections, with much attention was devoted to identifying people who were HIV‐infected and treating their intimate contacts. Because of the dire illnesses that people living with HIV developed, and the rapid growth of the epidemic, a sense of urgency led to funding dedicated siloed programmes, such as the NIH clinical trials networks, the PEPFAR initiative and the Global Fund, with scant consideration of concomitant STI that were frequently co‐prevalent. Yet at the same time, STI specialists who worked for decades with high disease burden populations found that support for their work was not expanding, and sometimes shrinking. The separation of HIV and STI support through categorical funding further impeded fruitful collaborations 9, 10.

Over the past decade, the situation has been altered dramatically because of the recognition that, although there are now tools to control the HIV epidemic, without addressing STI, their spread will accelerate, leading to widespread morbidity, complicating HIV control efforts 9, 10. Increases in congenital syphilis, expanding antimicrobial resistance in gonococci, and sexually transmitted Hepatitis C are three of many examples of how the global public health community has come to recognize that without addressing STI, the successes in the AIDS epidemic will be compromised. It is with that intent in mind that the International AIDS Society sponsored the STI 2018 pre‐conference in Amsterdam in July, 2018.

The two‐day meeting featured a variety of presentations that addressed the epidemiological, clinical, behavioural and structural issues that have driven the HIV and STI syndemics. Several key papers have been assembled for this special issue of the *Journal of the International AIDS Society* which summarize the key themes discussed at the conference, in order to inform readers about the current state of the science related to HIV and STI interactions and to discuss challenges ahead for a more integrated response to these syndemics. Taylor and Wi of the WHO describe the global epidemiology of STI spread, and notably, their paper points out that more than one million treatable STI occur daily across the globe, in addition to even larger numbers of chronic viral infections, like *Herpes simplex* and *Human papillomavirus* 11. Moreover, many of these infections are prevalent in areas of high HIV incidence, or among populations of greatest HIV risk, underscoring the need for combination approaches to address STI and HIV. Wi and WHO colleagues describe the current status of clinical management of STI globally, noting the issues related to the continued frequent use of empiric, syndromic management 12. The problems with this approach include mistreating vaginal discharges with inappropriate antibiotics and selecting for antibiotic resistance, and missing the high burden of asymptomatic infections such as chlamydia, especially in women. The paper challenges global public health leaders to strategize about how to provide point‐of‐care and state‐of‐the‐art nucleic acid amplification testing to make aetiologic diagnoses in resource‐constrained, but high disease burden, areas. It is notable that many countries that have active tuberculosis control programmes have platforms that can perform rapid molecular STI screening, but they are not being used for this purpose. More creative thinking about how to integrate these programmes, as well as how to lower the costs of reagents, may lead to wider access to appropriate management of STI.

Although STI are prevalent among the general population in resource constrained populations of the world, higher concentrations are often seen in key populations (KP) such as MSM, transgender women, people who inject drugs, sex workers and migrants. Mayer and Allan‐Blitz have summarized many of the factors that increase the KP STI risk, which include biological factors such as the increased susceptibility of anal mucosa to specific STI pathogens, behavioural factors such as depression and substance use leading to lack of self‐protective behaviours, structural factors such as punitive legal frameworks and culturally insensitive healthcare workers, resulting in avoidant healthcare behaviour 13.

This special issue also includes several papers focusing on the unique biological interactions of HIV and STIs. Cohen and colleagues summarize what is known about the biological interactions of HIV and STI before and after the advent of highly active antiretroviral therapy 14. Mwatelah and colleagues summarize the state of knowledge regarding HIV transmission in African women, who may have microbial ecological factors that increase their HIV risk, such as the low prevalence of protective vaginal lactobacilli, as well as socio‐epidemiologic factors such as the increased likelihood of choosing older partners who may already be HIV‐infected 15. Chow and colleagues summarize the current state of knowledge regarding extra‐genital STI 16. Their paper discusses some of the questions regarding the role of oro‐genital sex in potentiating the spread of gonorrhoea and chlamydia.

The next set of papers focuses on clinical issues that are emerging regarding HIV and STI. de Vries discusses the challenges that clinicians face in addressing STI in the current treatment‐as‐prevention era 17. Rojas Castro and colleagues discuss the patterns of STI that have been seen in individuals who use biomedical HIV prevention, that is, PrEP, and address the question of risk compensation versus risk maintenance when individuals who are at high risk for HIV/STI utilize interventions that can protect them against HIV but not STI 18. The topic of emerging infectious diseases is discussed by Nijmeijer and colleagues, focusing primarily on Hepatitis C but also discussing the potential of other agents that are not often thought of as being sexually transmitted to emerge when behavioural patterns change 19. Rietmeijer discusses the evolution of STI clinics in recent years, and their continued need to change in order to optimally co‐manage HIV and STI 20. Lastly, Rojas Castro and colleagues discuss the supreme importance of engaging affected communities in order to conduct effective clinical research, to translate science into clinical care, given that stigma is frequently associated with HIV and STI in populations who may have reasons to mistrust the beneficence of researchers and clinicians 21.

The intent of this report is to provide new data to interested readers, to hopefully stimulate further discussion. There remain many questions about optimal strategies to enhance the uptake of, and adherence to, antiretrovirals for treatment and prevention, while at the same time increasing diagnosis, treatment, and partner identification of people who are infected with STI. In the worst case scenario, in a world where antiretrovirals are not easily accessible, and/or the co‐factors that affect adherence are not addressed, and STI are not promptly diagnosed and treated, both epidemics could exacerbate each other, leading to a new dark era, which is an emerging reality in many countries in Eastern Europe and Central Asia. Hopefully, this will not be the case, and instead, the ability to offer sexually active people effective means to prevent them from acquiring or transmitting HIV will be incentive for them to come in for frequent STI screening, thereby leading to a mitigation of the spread of STI because of earlier diagnosis and partner notification. In this optimistic scenario, the synergism between the two epidemics could hopefully lead to fewer new STI and HIV infections, but in the short run, there will be substantial need for ongoing research, as well as professional and community education, in order to optimize the promising tools we currently can use.

## Competing interests

KHM and HdV have no competing interests to report.

## Authors’ contributions

KHM and HdV discussed the content and style of the paper. KHM drafted the paper, and HdV reviewed and edited the draft.
